# Joint association between ambient air pollutant mixture and pediatric asthma exacerbations

**DOI:** 10.1097/EE9.0000000000000225

**Published:** 2022-08-15

**Authors:** Jagadeesh Puvvula, Jill A. Poole, Sandra Gonzalez, Eleanor G. Rogan, Yeongjin Gwon, Andrew C. Rorie, Linda B. Ford, Jesse E. Bell

**Affiliations:** a Department of Environmental, Agricultural, and Occupational Health, College of Public Health, University of Nebraska Medical Center, Omaha, NE; b Division of Allergy and Immunology, Department of Medicine, University of Nebraska Medical Center, Omaha, NE; c Division of Public Health, Epidemiology and Informatics Unit, Department of Health and Human Services, Lincoln, NE; d College of Education and Human Sciences, University of Nebraska-Lincoln, Lincoln, NE; e Department of Biostatistics, College of Public Health, University of Nebraska Medical Center, Omaha, NE; f Asthma and Allergy Center, Bellevue, NE; g School of Natural Resources, University of Nebraska-Lincoln, Lincoln, NE; h Daugherty Water for Food Global Institute, University of Nebraska, Lincoln, NE

**Keywords:** Ambient air pollutants, Mixture, Pediatric asthma exacerbations

## Abstract

Exposure to air pollutants is known to exacerbate asthma, with prior studies focused on associations between single pollutant exposure and asthma exacerbations. As air pollutants often exist as a complex mixture, there is a gap in understanding the association between complex air pollutant mixtures and asthma exacerbations. We evaluated the association between the air pollutant mixture (52 pollutants) and pediatric asthma exacerbations.

**Method:**

This study focused on children (age ≤ 19 years) who lived in Douglas County, Nebraska, during 2016–2019. A seasonal-scale joint association between the outdoor air pollutant mixture adjusting for potential confounders (temperature, precipitation, wind speed, and wind direction) in relation to pediatric asthma exacerbation-related emergency department (ED) visits was evaluated using the generalized weighted quantile sum (qWQS) regression with repeated holdout validation.

**Results:**

We observed associations between air pollutant mixture and pediatric asthma exacerbations during spring (lagged by 5 days), summer (lag 0–5 days), and fall (lag 1–3 days) seasons. The estimate of the joint outdoor air pollutant mixture effect was higher during the summer season (adjusted-β_WQS_ = 1.11, 95% confidence interval [CI]: 0.66, 1.55), followed by spring (adjusted-β_WQS_ = 0.40, 95% CI: 0.16, 0.62) and fall (adjusted-β_WQS_ = 0.20, 95% CI: 0.06, 0.33) seasons. Among the air pollutants, PM_2.5_, pollen, and mold contributed higher weight to the air pollutant mixture.

**Conclusion:**

There were associations between outdoor air pollutant mixture and pediatric asthma exacerbations during the spring, summer, and fall seasons. Among the 52 outdoor air pollutant metrics investigated, PM_2.5_, pollen (sycamore, grass, cedar), and mold (*Helminthosporium*, *Peronospora*, and *Erysiphe*) contributed the highest weight to the air pollutant mixture.

**What this study adds**

This study evaluated the joint effect of 52 air pollutants with pediatric exacerbations upon adjusting for weather metrics, using the gWQS regression with repeated holdout validation. We observed associations between air pollutant mixture exposures and pediatric asthma exacerbations during the spring, summer, and fall seasons. Our findings suggest that PM_2.5_, pollen (sycamore, grass, cedar), and mold (*Helminthosporium*, *Peronospora*, and *Erysiphe*) played a crucial role in the joint association between air pollutant mixture and pediatric asthma exacerbations.

## Introduction

Asthma is a chronic airway inflammatory disease characterized by repeated episodes of wheezing, breathlessness, cough, and reversible airway obstruction that is often exacerbated by environmental exposures.^1,2^ In the United States, 46% of the population experience at least one exacerbation of disease per year, and the prevalence among children is 10% higher than in adults.^3^ During 2010–2018, approximately 88 per 10,000 children/year sought emergency care in the United States due to an exacerbation of asthma.^4^ Pediatric asthma incurs a severe economic burden on the US healthcare system, with a total direct cost of $5.9 billion per year.^5^

Asthma exacerbations are typically associated with environmental factors such as criteria pollutants (sulfur dioxide, nitrogen dioxide, carbon monoxide, ozone, particulate matter), aeroallergens (pollen, mold), and weather conditions (thunderstorms).^6–11^ Exposure to particulate matter with a diameter of less than 2.5 μm (PM_2.5_), sulfur dioxide, nitric oxides (NO), and ozone have each been identified to play an important role in pediatric asthma exacerbations.^12–14^ Unlike the other criteria pollutants, particulate matter is a complex mixture containing a number of viable and nonviable components including pollen fragments, bacterial products, metal, acids, and organic chemicals.^15^

Extensive efforts are emerging to characterize nonviable particulate matters such as metals, carbon (elemental, organic, black)^16^ and its associations with pediatric respiratory outcomes.^17–19^ Despite ample evidence on the associations between aeroallergens and pediatric asthma,^20–23^ there is limited information available that could be generalized at a community scale. Air pollutants often occur as a complex mixture, and evaluating associations between the numerous outdoor air pollutants and human health outcomes requires a multipollutant mixture analytic approach.^24^ Environmental mixture analysis techniques are ideal for toxin identification, mixture effect, and individual contribution.^25,26^ To the best of our knowledge, there has been one prior study focused on the combined effect of air pollutants (ozone, NO, and PM_2.5_) and pediatric asthma using a categorical joint effect model.^27^ However, the compound effect of pollen and mold along with criteria pollutants in the context of the compound effect modeling framework has not been explored. Mixture effect models play a crucial role in understanding the overall synergistic effect of air pollutants and identifying candidate pollutants among the air pollutants that contribute the most to triggering pediatric asthma exacerbations.

Thus, this study aimed to evaluate the association in time (0–5 lag days) between outdoor air quality mixtures of 52 air pollutants, including eight criteria pollutants, 27 pollen metrics, 17 mold metrics, and pediatric asthma exacerbations.

## Methods

### Study area

This study evaluated the association between the compound exposure of air pollutant mixture (criteria pollutants, pollen, and mold) and pediatric asthma exacerbations in Douglas County, Nebraska (NE), from 2016 to 2019. The associations were assessed by meteorological seasons (Winter: December–February [325 days]; Spring: March–May [364 days]; Summer: June–August [364 days]; Fall: September–November [360 days]). Douglas County is the most populated county in the state of Nebraska, with 29% of the state population concentrated in the county.^28^ Approximately 28% of the Douglas County population is under 19 years of age.^28^

### Pediatric asthma exacerbations

Data from emergency department (ED) visits was obtained from the Nebraska Hospital Information System (NHIS), maintained by the Nebraska Hospital Association. The NHIS database contains nonproprietary healthcare claims data (8,37I obtained directly from hospitals).^29,30^ This study includes ED visit data from 1 January 2016 to 31 December 2019 of children (age ≤ 19 years and living in Douglas County, NE) who visited an ED with a primary diagnosis of asthma exacerbation with an ICD-10 code of J45.x (4,195 ED visits). We excluded subjects diagnosed with exercise-induced bronchospasm (J45.990; 44 children). In total, 4,151 children were included. The study protocol was reviewed by the University of Nebraska Medical Center, Institutional Review Board, Protocol #0629-21-EP.

The pediatric population estimates for Douglas County, NE, were obtained from the 2019 American Community Survey (5-Year) data.^28^

### Air pollutant exposure

This study includes criteria pollutants (8), pollen (27), and mold (17) obtained from multiple sources. Daily observations of the air pollutants and weather data were obtained at the County scale.

### Criteria pollutants

We obtained the criteria pollutant measurements from the US Environmental Protection Agency (US EPA) ground monitoring station located at the Douglas County Health Center (AQS Site ID: 31-055-0019).^31^ The daily maximum observations of carbon monoxide (CO), NO, nitrogen dioxide (NO_*y*_), reactive oxides of Nitrogen (NO_*y*_-NO), sulfur dioxide (SO_2_), ozone (O_3_), and particulate matter (PM_2.5_ and PM_10_) were obtained from the samples obtained at an hourly scale. The metrics CO and O_3_ were measured as parts per million (PPM), NO, NO_y,_ NO_*y*_-NO, and SO_2_ were measured as parts per billion (PPB), and particulate matter (PM_2.5_ and PM_10_) was measured as micrograms per cubic meter.

### Aeroallergens (pollen and mold)

The aeroallergen data were obtained from the Asthma and Allergy Center, located in Bellevue, Nebraska (an adjacent suburb to Omaha). The aeroallergen monitoring station located in Bellevue, NE, is the only station in Nebraska accredited by the National Allergy Bureau.^32^ The aeroallergen monitoring station is located within a 15-mile radius of the Douglas County region. The pollen and mold samples were obtained from a sampling station located on the third story rooftop of the UNMC Bellevue Medical Center. The samples were collected using Rotorod Sampler Model 40 (1.52 × 1.52 × 32 mm). The sampling strategy involves the application of silicone grease to the rod and rotation of the retracting rod at a speed of 2,400 rpm for 1 minute in every 10-minute interval for 24 hours. The samples were then collected daily at 7 am by placing the retractable rod on the stage adapter. The samples were stained using Calberla’s stain and counted at 40X under the microscope. The pollen and mold counts were estimated as particles per 3.12 m^3^ of air.

Among the 44 aeroallergens measured at the Asthma and Allergy Center, 27 variables correspond to pollen and 17 to mold. The pollen metrics could be further clustered into 17 tree pollen, 7 weed pollen, 1 grass pollen, and 1 unknown pollen measurement.

### Weather indicators

Daily observations of maximum temperature, precipitation, average wind speed, and wind direction (fastest 5-second wind direction) data were obtained from the Global Historical Climatology Network (GHCNd).^33^ These observations were retrieved from the monitoring station located at the Omaha Eppley Airfield (Station ID: USW00014942). The daily temperature was measured in Celsius, precipitation in millimeters, wind speed in miles/hour, and wind direction in degrees.

### Missing data imputation

The missing air pollutant observations (~6%) were imputed using Probabilistic Principal Component Analysis (PPCA).^34,35^ Air quality and weather metrics were included as loading variables in the PPCA. We estimated the root mean square percentage error (RMSPE) by performing cross-validation to estimate the prediction error introduced during the imputation and observed that the RMSPE values ranged between 4% and 12% per metric. The analytic dataset (1,413 days) contains pediatric asthma exacerbation count, air pollutants (52 variables), and weather metrics (six variables), measured on a daily scale.

### Statistical analysis

To assess the correlation across the 52 air pollutants included in this study, we estimated the Spearman correlation coefficients stratified by four seasons. Statistically significant correlations were considered for the correlation coefficients with a *P* value less than 0.05. The association between the compound exposure of air pollutant mixture and pediatric asthma exacerbation emergencies was evaluated using generalized Weighted Quantile Sum (gWQS) regression with a repeated holdout.^36,37^ The regression model was implemented using a mixture of 52 air pollutants constructed on a quantile scale as an exposure variable. The associations were evaluated using the uncontrolled interrupted time-series (UITS) design to preserve the temporal trend that allowed us to assess the lagged exposure-response associations by the season. The primary outcome was the daily count of pediatric asthma-related ED visits. We included daily temperature, precipitation, wind speed, and wind direction in the statistical model to adjust for the potential confounding effect of weather metrics on pediatric asthma. The association between air pollutant mixture and pediatric asthma exacerbation-related ED visits was evaluated. The gWQS regression model was implemented assuming that the air pollutants would have a positive association with pediatric asthma exacerbations due to its conditional homogeneity. These associations were presented as the same-day exposure-response relationship (lag_0_) and delayed associations where the air pollutant mixture exposure was evaluated for potential associations with pediatric asthma exacerbations that could be delayed by 1–5 days.

The analytic dataset was split into training (40%) and validation (60%) data. We considered 100 bootstrap samples per iteration and repeated them for 100 iterations using repeated holdout to stabilize the effect estimates. The model output provided a mean effect estimate of the outdoor air pollutant mixture and a 95% confidence interval (CI). Additionally, the contribution of each air pollutant included in the WQS mixture was reported as a median percentage and its 95% CI. All the analyses were conducted using statistical software R version 3.5.1.

## Results

In Douglas County, NE, there were 15.8 (age-adjusted) asthma-related ED visits per 10,000 children/year. Over the four years, the age-adjusted rate of ED visits was higher in 2016, with an estimate of 17.96 ED visits per 10,000 children/year (Table 1). During the study period, the rate of pediatric asthma exacerbations was consistently higher during the spring and fall seasons. The annual median crude rate of ED visits was higher among male children of age under 5 years (102 per 10,000 children/year) and children of age between 5 and 9 years (103.8 per 10,000) compared with children of age 10–14 years (63 per 10,000) and 15–19 years (29 per 10,000). A similar trend was not observed with the female population (S1A and S1B; http://links.lww.com/EE/A197).

**Table 1.** Rate of pediatric asthma ED visits per season and year per 10,000 children.

Year	Season^a^									
	Winter		Spring		Summer		Fall		Overall^b^	
	AAR	CR	AAR	CR	AAR	CR	AAR	CR	AAR	CR
2016	3.23	13.64	5.06	20.80	3.18	10.02	6.38	26.10	17.96	73.56
2017	3.22	13.27	4.50	18.44	3.27	13.32	4.83	19.75	15.83	64.78
2018	1.86	7.60	4.36	17.82	3.57	14.64	4.13	17.01	13.93	57.05
2019	3.03	12.27	4.98	20.50	2.66	10.90	4.80	19.50	15.47	63.16

^a^Per season (3 months).

^b^Per year.

AAR indicates age-adjusted rate; CR, crude rate.

There were 2–4 ED visits per day in all four seasons during the study period. There were 172 days (12.17%) during the study period (1,413 days) without any ED visits for pediatric asthma exacerbation. The summer season had the highest number of days (71 days, 19.50%) without any ED visits, followed by winter (53 days, 16.30%), fall (25 days, 6.94%), and spring (23 days, 6.31%). The highest number of ED visits for asthma exacerbations was during the spring and fall season.

We identified statistically significant positive correlations between ozone and mold counts during the winter (r = 3%–12%; *P* < 0.05); between the 17 mold species (r = 12%–83%; *P* < 0.05) (S2A; http://links.lww.com/EE/A197). In spring, we found significant positive correlations between ozone and mold (r = 0.8%–26%; *P* < 0.05); tree pollen (hickory, walnut, pine) and mold (r = 0.2%–56%; *P* < 0.05) (S2B; http://links.lww.com/EE/A197). In the summer, there were statistically significant negative correlations between ozone and mold (r = 0.3%–21%; *P* < 0.05), and significant positive correlations between pollen counts from tree and grass (r = 12%–47%; *P* < 0.05) (S2C; http://links.lww.com/EE/A197). In the fall season, there were significant negative correlations between mold, pollen (weed and grass) and criteria pollutants (Nitrogen oxides) (r = 15%–36%; *P* < 0.05). In addition, there were significant positive correlations between pollen (weed, grass) and mold count (r = 3%–69%; *P* < 0.05) during fall season (S2D; http://links.lww.com/EE/A197).

The association between outdoor air pollutant mixture and pediatric asthma exacerbations was evaluated on a seasonal scale. We observed associations between the outdoor air pollutant mixture and pediatric asthma exacerbations during the spring, summer, and fall seasons. We observed statistically nonsignificant associations between the air pollutant mixture and pediatric asthma exacerbations during the winter season. Hence, we emphasized on the statistically significant associations observed during the spring, summer, and fall seasons.

**Figure 1.**
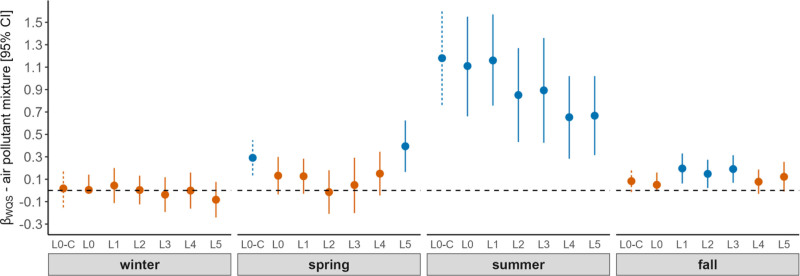
Association between outdoor air pollutant mixture and pediatric asthma exacerbations. The *y* axis represents the beta coefficient (effect estimate) of outdoor air pollutant mixture. The *x* axis represents seasons of a year. The CIs of the crude model are represented using dotted line and the adjusted model using solid line. Adjusted model includes temperature, precipitation, wind speed and direction as covariates. Effect estimates with statistically significant association were represented using blue and statistically nonsignificant associations were represented using orange. The effect estimates and 95% CIs were generated using qWQS regression with 100 repeated holdouts. L0-C represents the crude effect estimate with the same-day exposure-response association. L0–L5 are the adjusted effect estimates, where “0” represents the same day association and 1–5 represent the exposure-delayed response associations ranging from 1 to 5 days.

**Figure 2.**
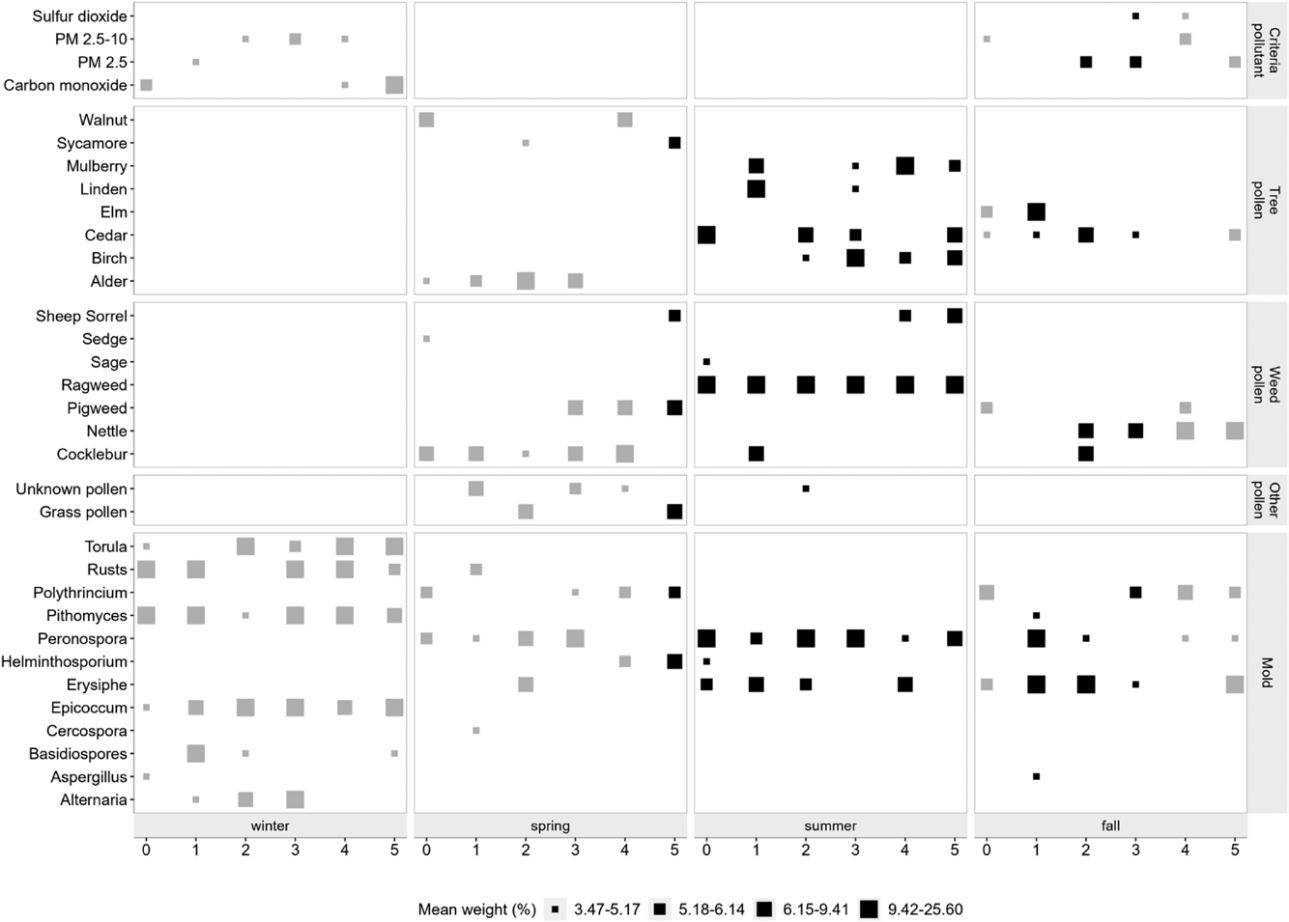
Contribution of individual pollutant to the air pollutant mixture weights. The *x* axis represent the lag period (0–5 days) by season. The *y* axis represent individual air pollutants contributed to the mixture effect. The size of the squares represents the percent mean weight of the pollutant contributed to the pollutant mixture. Larger the size of the square represents higher weight. The color of each square represents if the pollutant weight obtained from a model with statistically significant associations (Gray, nonsignificant; black, significant associations). Pollutant weights were obtained from the gWQS regression with 100 repeated holdouts.

We found delayed (lag_5-days_) associations between air pollutant mixture and asthma exacerbations during the spring season. Namely, in the spring season, three deciles increase in the air pollutant mixture was associated with about 1 asthmatic ED visit per day that lagged by 5 days postexposure (adjusted β_WQS_ 0.40, 95% CI: 0.17, 0.63) (Figure 1). Among the air pollutants included in this study, tree pollen (sycamore), grass pollen, and mold (*Helminthosporium, Polythrincium*) contributed the highest weights to the pollutant mixture effect during the spring season (Figure 2). Sycamore and grass pollen are typically seen during April and May, with a peak pollen count in late April. At the same time, the *Helminthosporium* and *Polythrincium* were observed during most of the days in the spring season (Table 2, (S3 and S4; http://links.lww.com/EE/A197).

**Table 2.** Descriptive statistics of pollutants with a higher contribution to the mixture effect.

Season	Pollutant	Mean weight	% Days nonzero values	Air pollutant concentration	
				Median (IQR)	Mean (SD)
Spring	Grass pollen^a^	0.073	24.53	0 (0)	10.34 (37)
	Helminthosporium^a^	0.056	11.17	0 (0)	4.65 (20.1)
	Sycamore^a^	0.052	25.89	0 (0-1)	4.91 (23.4)
Fall	Erysiphe^a^	0.045–0.138^c^	13.61	0 (0)	12.46 (42.6)
	Cedar^a^	0.044–0.064^c^	30.83	0 (0-1)	5.60 (25.2)
	PM2.5^b^	0.056–0.057^c^	100	13 (10-17)	13.60 (5.49)
Summer	Peronospora^a^	0.046–0.157^c^	16.30	0 (0)	15.40 (49.3)
	Ragweed^a^	0.107–0.145^c^	28.53	0 (0-2)	39.38 (133)

^a^measured asparticles per 3.12 m^3^ of air.

^b^Measured as micrometer per cubic meter.

^c^Range of mean percent weights contributed across the lag period (1–3 days for fall season and 0–5 for summer season).

IQR indicates interquartile range; SD, standard deviation.

We observed that associations between air pollutant mixture and pediatric asthma exacerbations lagged by 1–3 days in the fall season. In the fall season, five deciles increase in the air pollutant mixture was associated with approximately 1 ED visit/day (adjusted β_WQS_ range 0.15–0.20), lagged by 1–3 days (S1; http://links.lww.com/EE/A197; Figure 1). Among the air pollutants, mold (*Erysiphe*), tree pollen (cedar), and PM_2.5_ were higher and commonly contributed to the air pollutant mixture effect over lag 1–3 days during the fall season. During the study period, we observed the peak spore count of *Erysiphe* during September and followed a declining trend until November. Although cedar pollen is recognized to commonly peak between February and April, we observed cedar pollen starting from September until May (S3; http://links.lww.com/EE/A197).

During the summer season, we observed associations between air pollutants and pediatric asthma exacerbations (lag 0–5 days). There is a declining trend of effect estimates starting from the same day associations to the effect lagged by 5 days. Focusing on the same-day exposure-outcome associations, each decile increase in the outdoor pollutant mixture was associated with about 1 ED visit per day (adjusted β_WQS_ 1.11, 95% CI: 0.66, 1.55) increase in pediatric asthma exacerbations. Mold (*Peronospora*) and weed pollen (ragweed) had higher contributions and commonly contributed to the mixture effect over a lag of 0–5 days. The effect estimate decreased up to 20% from the same-day exposure-response compared to the associations lagged by 2–3 days. Additionally, the effect estimate declined up to 40% from the same day exposure compared to the association lagged by 4–5 days. We observed ragweed pollen during the study period starting from July, with the annual peak counts during August. In contrast, *Peronospora spore* counts were observed throughout the year, with an annual peak spore count between July and September.

## Discussion

This study evaluated the association between outdoor air pollutant mixture and pediatric asthma exacerbation-related emergency visits in Douglas County, NE. The seasonal-scale associations were analyzed using daily scale data over 4 years (2016–2019). This study evaluated the same-day (lag_0_) exposure-response and delayed associations that lagged up to 5 days. This study included 52 air pollutants that were categorized as criteria pollutants, pollens, and molds, as a mixture and were included in the gWQS regression model using a quantile scale. Additionally, the joint association of air pollutant mixture was estimated upon adjusting for temperature, precipitation, wind speed, and wind direction.

The rate of pediatric asthma exacerbation-related ED visits peaked during the spring and fall season. The seasonal trend of the pediatric asthma exacerbations that required ED visit aligned with the results from a national scale study that reported higher asthma rates during the March–May and September–November periods.^38^ Additionally, Rodrigues et al.^39^ reported a similar seasonal pattern of asthma-related hospitalizations among children in Lisbon, Portugal, suggesting the seasonal relevance corresponding to the beginning of the school year. The overlap between increased pediatric asthma exacerbations and the beginning of the school year could be due to an extended outdoor exposure window among children 4–19 years or older.^40^

This study observed a positive association between outdoor air pollutant mixture and pediatric asthma exacerbations during the spring, summer, and fall seasons. The exposure-outcome associations during the summer season were consistent with a lag of 0–5 days. Whereas during the spring season, the associations of pollutant mixture levels to pediatric asthma exacerbations lagged by 5 days, in the fall season, there was a lag of 1–3 days. These associations during the summer season could be explained by total outdoor time spent, which is heavily influenced by weather conditions.^41,42^ Meng et al.^43^ reported that children are more vulnerable to total air pollutant exposure during non-winter days than winter. In this study, there were lower criteria pollutant concentrations and null values for aeroallergen counts for the majority of the winter days. This could be also due to logistical issues in measuring aeroallergens during the winter days with snow and ice. In addition, we suspect that harsh winter weather conditions preclude outdoor activities by children and thus minimize exposure.

Pollen from grass and plants (sycamore), as well as mold (*Helminthosporium*), contributed higher weights to the outdoor pollutant mixture in the spring season. In the fall season, tree pollen (cedar), mold (*Erysiphe*), and PM_2.5_ consistently contributed over lag 1–3 days to the outdoor air pollutant mixture. In the summer season, mold (*Peronospora*) and weed pollen (ragweed) consistently contributed to the outdoor air pollutant mixture associated with pediatric asthma exacerbations with a lag period ranging from 0 to 5 days.

A few case-crossover studies reported that exposure to outdoor air pollutants such as pollen (tree [birch], weed, and grass), mold (*Alternaria*, *Cladosporium*, *Aspergillus*, *Helminthosporium*, and *Penicillium*), and criteria pollutants (particulate matter [PM_2.5_] and ozone) are associated with an increase in pediatric asthma exacerbations.^44–52^ Additionally, Cox et al.^53^ reported that indoor mold exposures have also been associated with wheezing among children. Our results shared some common attributes with the existing literature,^44–51^ by demonstrating the association between outdoor air pollutants (mold and criteria pollutants) and pediatric asthma exacerbations.

Weed pollen exposure as a mixture was associated with an increase in pediatric asthma exacerbations, whereas a stratified analysis focused on the ragweed exposure was reported to be associated with a decline in pediatric asthma exacerbations.^44,45,52^ In contrast, a few studies reported that ragweed exposure is associated with increased respiratory symptoms (wheezing, shortness of breath, and cough) and asthma exacerbations among children.^54,55^ These findings suggest conflicting associations between ragweed exposure and pediatric asthma exacerbations reported in the literature. Our results showed that ragweed consistently contributed to the outdoor air pollutant mixture effect that is associated with an increase in pediatric asthma exacerbations during the summer season.

Additionally, tree pollen contributed to the air pollutant mixture during the spring and fall seasons. Even though trees typically pollinate during the spring season, we observed about 31% of days during the fall season with cedar pollen. A similar pattern was observed for cedar pollen measurements in different cities in the great plains region (Texas, Missouri, Oklahoma).^56^ Lo et al. reported cedar pollen counts that could be clustered into two seasons (January–March and September–December) that were observed in Missouri (Springfield), Oklahoma (Tulsa, Oklahoma City), and Texas (Waco, Austin, Houston, College Station, San Antonio). Our results suggest that cedar pollen contributed to the air pollutant mixture during the fall season (mean pollen concentration: 5.6; SD: 25.2), which reflects pollen counts 56 times less than the pollen counts during the spring season (mean: 311; SD: 1,493).

Asthma, one of the most common causes of morbidity and hospitalizations in childhood, is a complex disease influenced by many genetic factors and environmental factors including aeroallergens, pet dander, dust mite, molds, viral infections, and indoor and outdoor air pollution.^57–60^ Several of these environmental agents mediate disease consequences through disrupting the integrity of the airway epithelium and release of alarmins including thymic stromal lymphopoietin (TSLP), interleukin (IL)-33, and IL-25.^61^ These events trigger activation of mast cells, type-2 innate lymphoid cells (ILC2), T helper type 2 (Th2) cells, and eosinophilic recruitment and further release of Th2 cytokine (i.e., IL-4, IL-5, IL-9, IL-13).^61^ Specific immunoglobulin-E (IgE) to outdoor aeroallergens typically develops after the age of 3 years,^62^ the aeroallergens demonstrated in our study of grass pollen, tree pollen (sycamore, cedar), weed pollen (ragweed), and mold (*Helminthosporium*) are associated with allergic and asthmatic disease.^63^ Future studies evaluating allergenicity of the molds *Erysiphe* and *Peronospora* are warranted to understand their potential role in influencing allergic and asthma diseases.

This study has several strengths, such as utilizing robust outdoor pollutant (criteria pollutants, pollen, and mold) data at a daily scale and evaluating exposure-response associations using a mixture effect framework. In the context of air quality and human health outcomes research, most studies considered criteria pollutants as exposure. The substantial focus on criteria pollutants could be due to the US EPA Clean Air Act,^64^ which requires monitoring and reporting of criteria pollutants. As a result of the US EPA Clean Air Act, there are more than 4,000 air quality monitoring stations that measure hourly or daily levels of criteria pollutant measurements that are made publicly accessible at the US EPA Air Quality System database.^64^ The continuous monitoring and accessibility of criteria pollutant data could be one of the reasons that motivated researchers on evaluating health outcomes associated with criteria pollutants. As of 2021, there are about 85 aeroallergen measuring stations in the United States that collect pollen data at least 4 days/week.^32^ The availability of criteria pollutant and aeroallergen (pollen and mold) measurements at a daily scale allowed us to conduct a comprehensive study evaluating the association between air pollutants as a mixture and pediatric asthma exacerbations in Douglas County, NE. Similar study could be conducted using time-stratified case-crossover design to adjust for confounders such as age and gender. However, in this study, we followed the UITS design to preserve temporal trend to assess the lagged associations.

Our study also has limitations as the exposure data were based on outdoor monitoring stations, and the effect estimates are subjective to nondifferential misclassification of the exposure. Few studies compared the air pollutant exposure measured at a monitoring station to the personal exposures, found strong correlations for PM_2.5_, and sulfate concentrations that included the elderly population^65–68^ and PM_10_ exposure that included children.^69^ As the comparison between outdoor air pollutants and personal exposures is limited to particulate matter, there is a gap in understanding the dynamics of aeroallergens (pollen/mold). We did not consider an effect of single pollutant as our main contribution of this article to assess the joint association between air pollutant mixture (criteria pollutants, pollen, and mold) and pediatric asthma exacerbation-related ED visits. However, pollutants with higher weights from our results using air pollutants as a mixture overlapped with the findings from the single pollutant model conducted by Gonzales-Ramirez et al.^70^

Our results were also not standardized for population vulnerabilities such as poverty, race/ethnicity, and education, which could play a key role in differential exposure misclassification. We did not account for indoor exposure to house dust mites, cockroaches, pet dander (dog/cat), and smoking (primary/secondary), resulting in an unmeasured confounding effect. Due to convergence issues in the statistical model, we did not conduct stratified analysis to evaluate the role of confounding, interaction, or effect modification due to age, gender, body mass index, and co-morbidities associated with asthma exacerbations. Our study results are influenced by exposure misclassification, effect modification, and unmeasured confounding, violating exchangeability; therefore, our results do not infer causal relationships. Further cohort-based studies exploring the controlled direct effect, reference/mediated interaction, and pure indirect effect are necessary to establish a causal relationship between outdoor pollutants and pediatric asthma. A strength of this study was understanding the joint association between 52 air pollutants as a mixture and pediatric asthma exacerbations, future studies may also be warranted to explore interaction terms between air pollutants that contributed to higher weights. Additionally, climate change was identified to alter the aeroallergen season length and timing.^71,72^ As we observed that aeroallergens played a crucial role in pediatric asthma exacerbations, future studies evaluating asthma exacerbations in the context of climate change could minimize unmeasured biases.

## Conclusion

To our knowledge, this is the first study to evaluate the association between air pollutant mixture and pediatric asthma exacerbations. Our findings shared attributes with the existing literature based on single pollutant exposures. As criteria pollutants (influenced by the number of industries, motor vehicles) and aeroallergen levels (due to dominance of tree species, weather conditions) vary by geographic area, our findings have substantial public health implications in the Great Plains region. Identifying air pollutants at the household level and mitigating potential sources associated with indoor pollutants could substantially improve respiratory health among children.

## Conflicts of interest statement

The authors declare that they have no conflicts of interest with regard to the content of this report.

## Acknowledgments

We would like to acknowledge Michelle Kelly from The Asthma & Allergy Center, Bellevue, NE, for her assistance in providing the aeroallergen data. We would also like to thank the Claire M. Hubbard Foundation, Robert B. Daugherty Water for Food Global Institute at the University of Nebraska, and the University of Nebraska Medical Center Graduate Studies for their support.

## Supplementary Material


