# Supplementary material for: Joint association between ambient air pollutant mixture and pediatric asthma exacerbations
Source: Environ Epidemiol. 2022 Aug 15;6(5):e225. doi: 10.1097/EE9.0000000000000225 (PMC9556053; doi:10.1097/EE9.0000000000000225)
Supplement: Supplementary file 1 [file ee9-6-e225-s001.pdf]

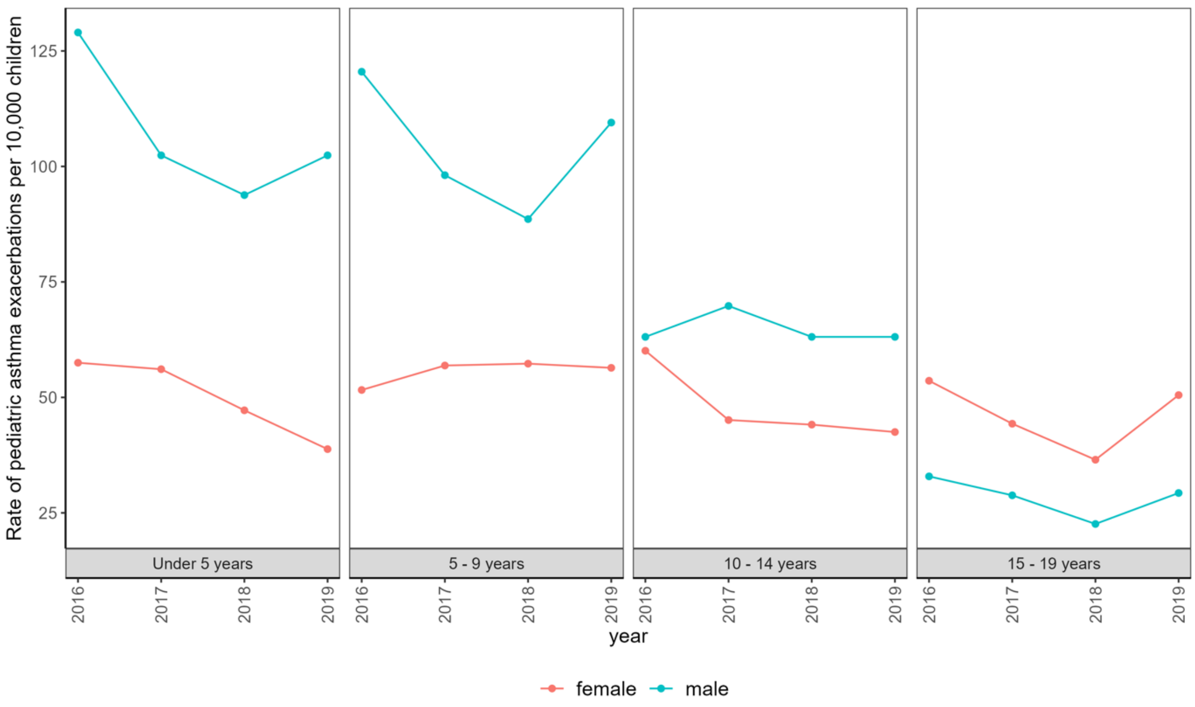

S 1A. Rate of pediatric asthma exacerbations per 10,000 children stratified by age groups and gender (2016-2019).

S 1B. Number of pediatric asthma exacerbations per age group, gender, and year

Year of ED visit	Age group (in years)			
	**Male**			
Year of ED visit	Under 5	5-9	10-14	15-19
2016	286	253	131	64
2017	227	206	145	56
2018	208	186	131	44
2019	227	230	131	57
	**Female**			
Year of ED visit	Under 5	5-9	10-14	15-19
2016	123	107	116	103
2017	120	118	87	85
2018	101	119	85	70
2019	83	117	82	97

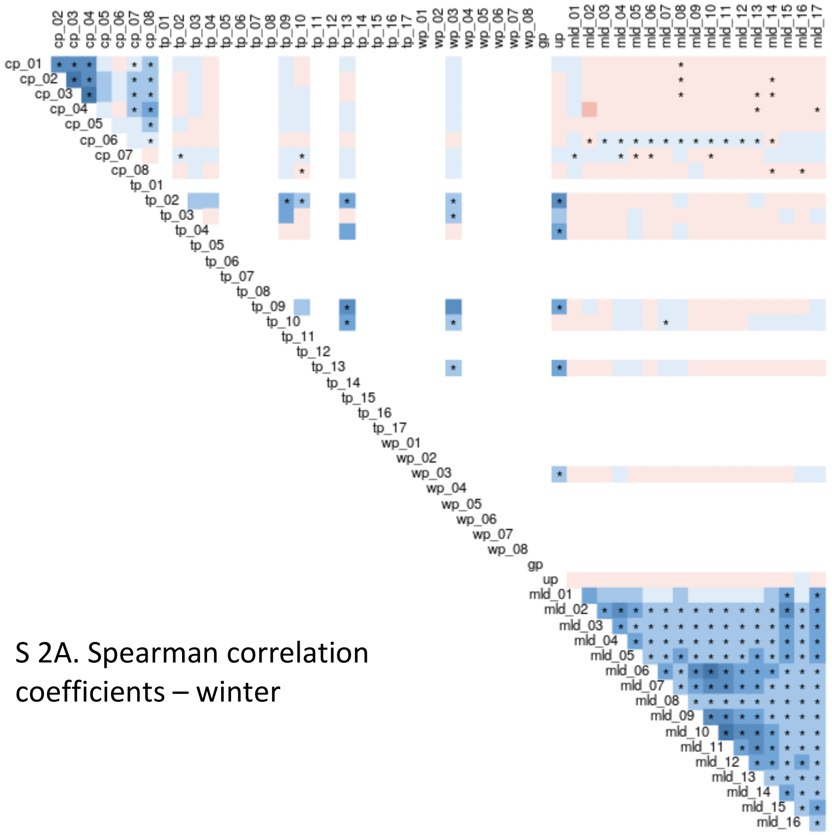

S 2A. Spearman correlation coefficients – winter

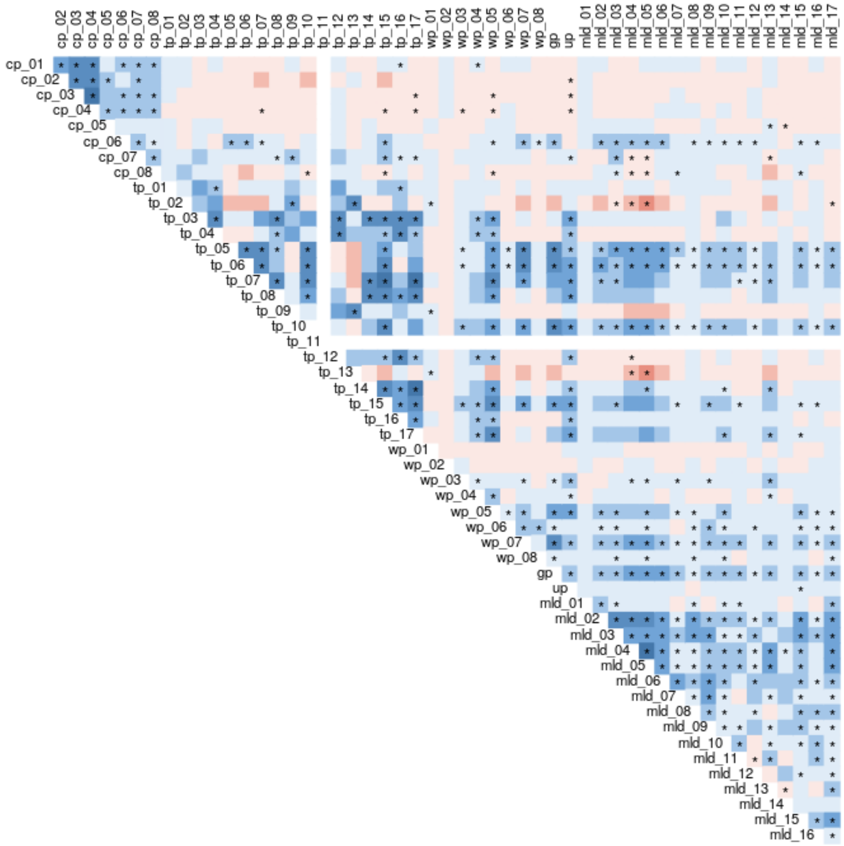

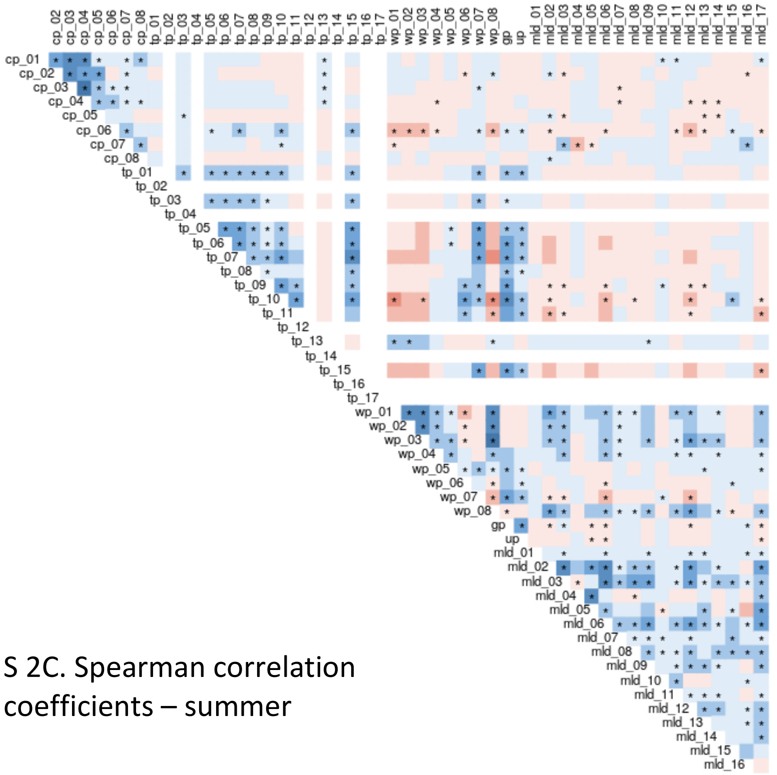

S 2C. Spearman correlation coefficients – summer

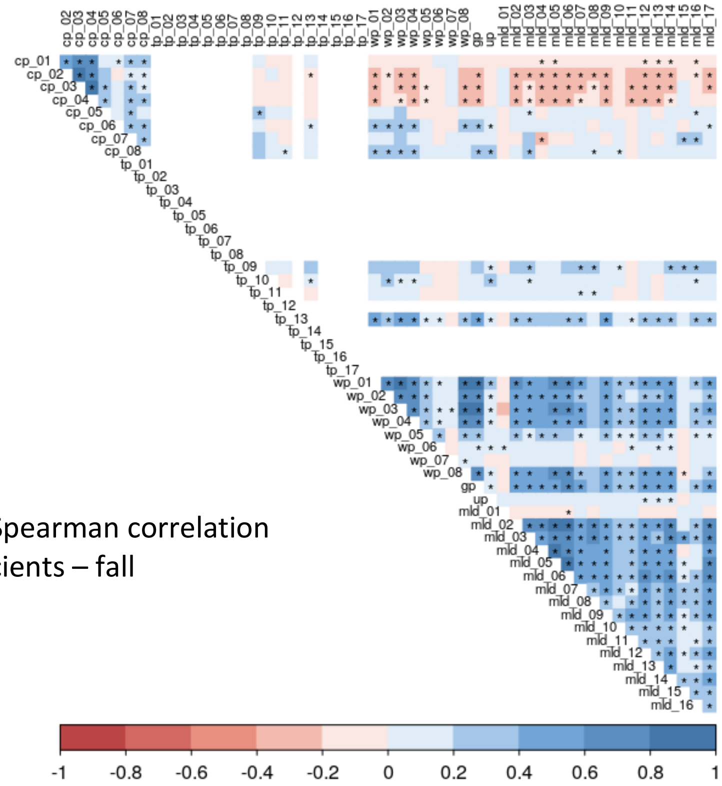

S 2D. Spearman correlation coefficients – fall

S 2A-D. Correlation between air pollutants during winter season. The correlation coefficients were estimated using Spearmen method and the combination of air pollutants with statistically significant correlation (p-value<0.05) are represented using asterisk sign. cp-Criteria pollutants, tp-tree pollen, wp-weed pollen, gp-grass pollen, up-unknown pollen, and mld-mold. The abbreviations for the air pollutants are available at: https://github.com/jagadeeshpuvvula/air_quality_asthma/blob/main/06.3_abbreviations

S 3. Weekly mean air pollutant levels by season

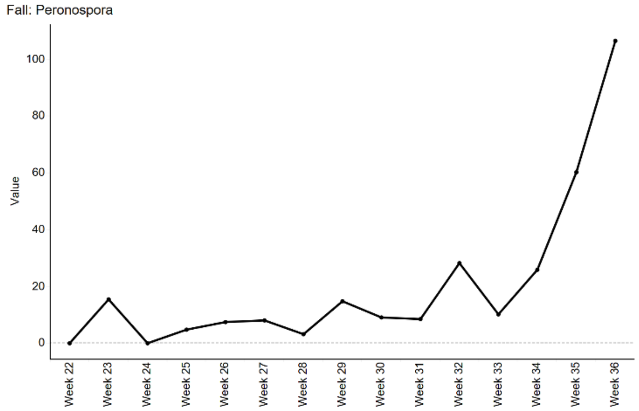

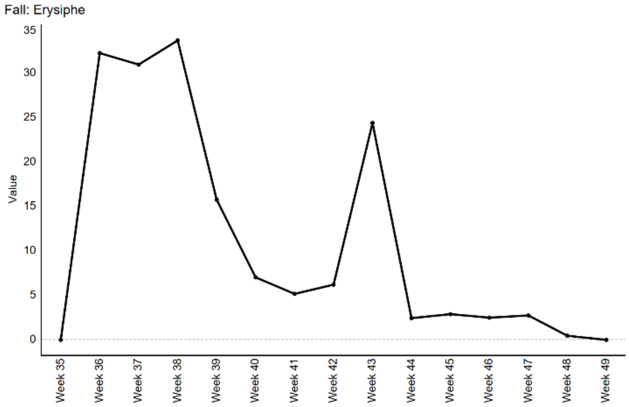

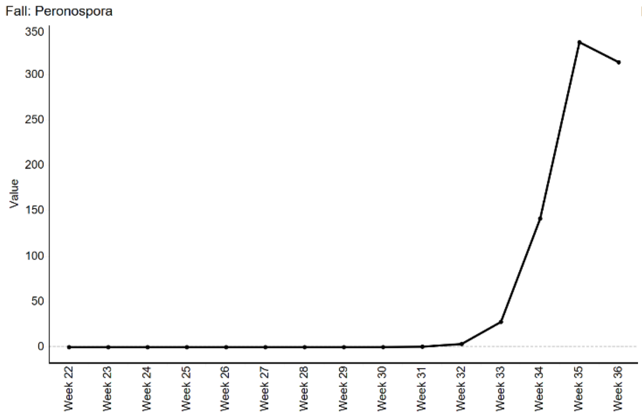

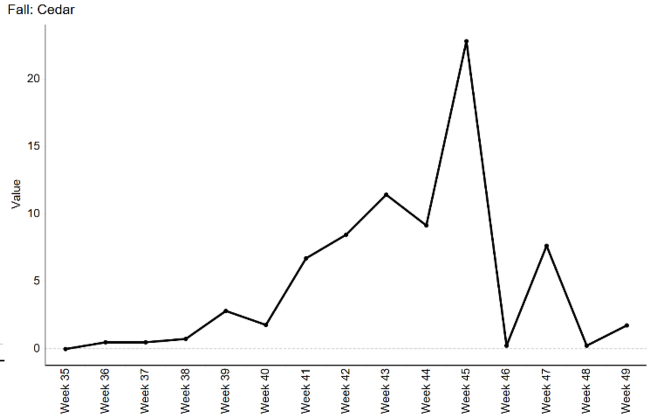

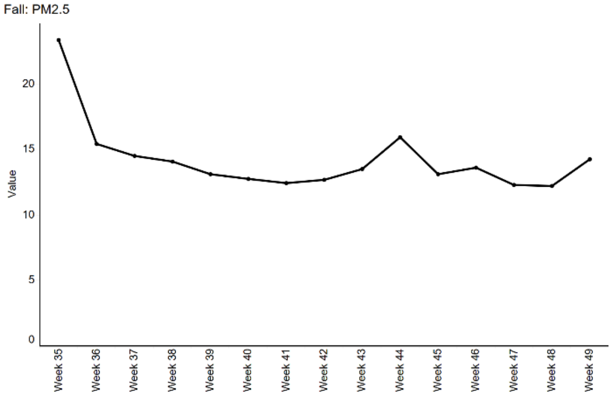

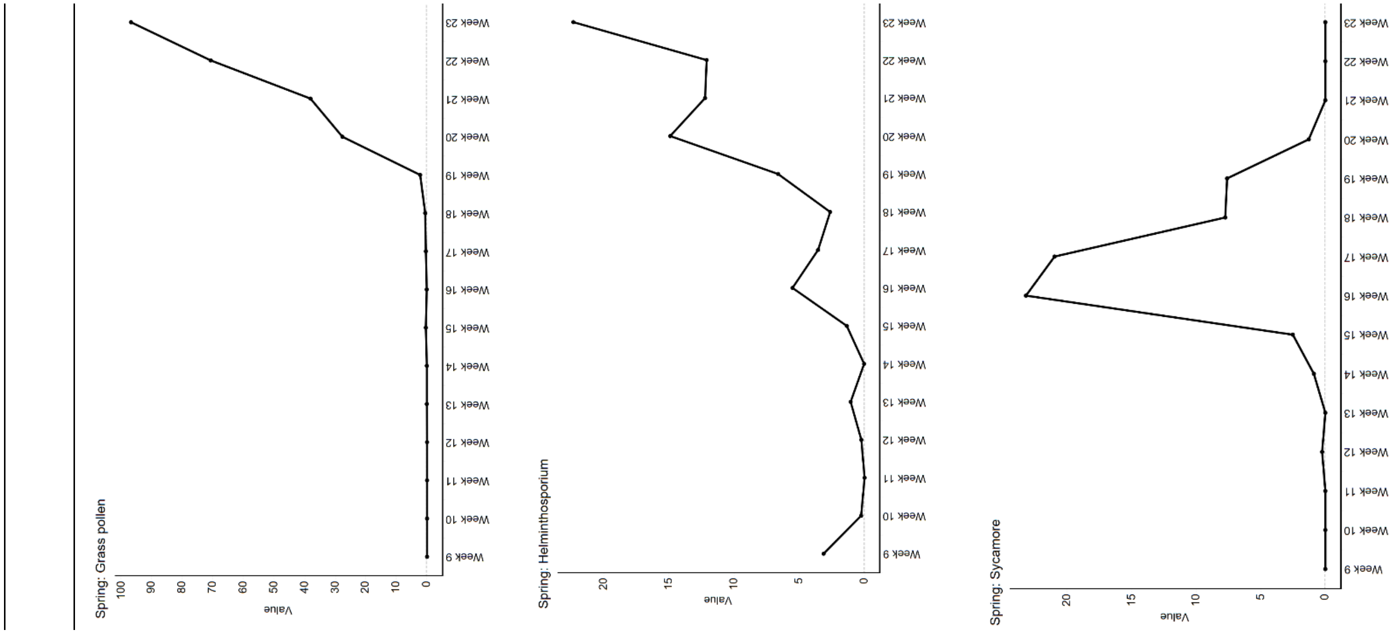

S 4. Descriptive of pediatric asthma exacerbations, criteria pollutants, pollen, and mold, stratified by season. The table includes percent of days with non-zero values, mean, standard deviation (SD), median, 25th percentile (Pct25) and 75th percentile (pct75). The table as a pdf at available at: https://github.com/jagadeeshpuvvula/air_quality_asthma/blob/main/summary.pdf
